# Genetic Associations of Functional Longevity With Sexual Precocity and Growth Traits in Nellore Beef Cattle

**DOI:** 10.1111/jbg.70065

**Published:** 2026-08-03

**Authors:** Dalila Ribeiro dos Santos, Gabriele de Souza Silva Fernandes, Arione Augusti Boligon, Mário Luiz Santana, Gilberto Romeiro de Oliveira Menezes, Denise Rocha Ayres, Annaiza Braga Bignardi, Rodrigo Junqueira Pereira

**Affiliations:** ^1^ Grupo de Melhoramento Animal de Mato Grosso (GMAT) Universidade Federal de Rondonópolis Rondonópolis MT Brazil; ^2^ Departamento de Zootecnia Universidade Federal de Pelotas (UFPEL), Campus Universitário Capão Do Leão Capão do Leão RS Brazil; ^3^ Embrapa Gado de Corte Campo Grande MS Brazil

**Keywords:** productive life, random regression, stayability

## Abstract

Functional longevity (FL) is a complex trait that integrates many attributes directly contributing to the successful retention and productivity of a cow within the herd. Consequently, improving FL in beef cattle increases herd profitability. This study aimed to estimate the genetic associations between two alternative FL definitions and traits related to sexual precocity and growth in Nellore cattle from data comprising 2,607,400 calving records. Two FL traits, evaluated from 27 to 135 months of age, were analysed using random regression animal models: the ability to remain in the herd, evaluated as a binary trait (FLSTAY), and the cumulative number of calvings up to the predefined standard ages (FLNC). Approximate genetic correlations among FL measures, age at first calving (AFC), weaning weight (WW), and yearling weight (YW) were estimated using Calo's method based on the estimated breeding values (EBVs) of individuals with an accuracy of at least 0.60 for both traits. Heritability estimates for FLNC ranged from 0.020 (95% highest posterior density [HPD]: 0.018 to 0.022) to 0.075 (95% HPD: 0.067 to 0.084) and were consistently higher than those for FLSTAY, which ranged from 0.004 (95% HPD: 0.004 to 0.005) to 0.045 (95% HPD: 0.039 to 0.051) across all evaluated ages. The approximate genetic correlations between FLSTAY and FLNC were positive and high (≈0.90) throughout the productive lifespan. Approximate genetic correlations between AFC and both FL measures were favourable, exhibiting a high magnitude at 27 months of age (≈−0.75) and gradually decreasing to values around −0.25. Approximate genetic correlations between WW (direct effect) and FL traits were favourable, starting high at 27 months (≈0.58) and gradually decreasing to 0.06 for FLNC and 0.15 for FLSTAY at older ages. Approximate genetic correlation estimates between WW (maternal effect) and both FLSTAY and FLNC were also favourable (≈0.20) across ages. YW followed a similar trend, showing favourable genetic correlations of ≈0.28 at 27 months that decreased to ≈0.10 near 75 months. Both FL traits exhibit sufficient genetic variability for long‐term selection. However, FLNC is recommended over FLSTAY as a selection criterion due to its consistently higher heritability. The favourable genetic associations demonstrate no antagonism between functional longevity and standard growth or sexual precocity traits, supporting the implementation of FLNC as a feasible alternative to traditional single‐measure stayability in Nellore breeding programs.

## 1 Introduction

Reproductive traits play a fundamental role in determining the economic sustainability of beef cattle herds, particularly when associated with the lifetime survival of a cow (Catrett et al. 2024; Santana et al. 2013). Increasing cow longevity reduces the need for replacement heifers, thereby creating additional opportunities for voluntary culling based on individual productivity (Martinez et al. 2004a). Consequently, cows with extended longevity tend to be more profitable, as they enable a more efficient recovery of the initial capital invested in their rearing (Garcia et al. 2015).

Historically, stayability has been the most common longevity measure used in beef cattle breeding programs. Traditional stayability is defined as the probability of a cow remaining in the herd up to a specific age and calving at least a specified number of times, provided she is given the opportunity to reach that benchmark (Carvalho et al. 2025; Ramos et al. 2020; Silva et al. 2018). Despite its widespread adoption, traditional stayability presents limitations for selection. The primary drawback of its inclusion in selection criteria is the delayed recording of phenotypic data, which increases the generation interval and reduces annual genetic gain (Jamrozik et al. 2013). Furthermore, stayability is typically expressed as a binary trait. This non‐normal distribution poses inherent challenges to genetic evaluations (Costa et al. 2019; Pereira et al. 2025) and frequently fails to differentiate cows based on their actual productive performance (Passafaro et al. 2024; Ramos et al. 2021; Silva et al. 2018).

To overcome the inherent limitations of traditional stayability, researchers have investigated alternative traits that can be recorded earlier in a cow's life. One approach involves shifting the reference age to earlier benchmarks, such as 48 or 52 months, which could reduce the generation interval and increase the rate of genetic gain (Pereira et al. 2025; Ramos et al. 2020). However, this approach still faces difficulties in properly differentiating highly productive cows from those with average performance. Other initiatives have proposed single‐measure traits based on the cumulative number of calvings up to a certain age to discriminate cows according to their productive performance (Brzáková et al. 2019; Venot et al. 2013; Neves et al. 2012; Martinez et al. 2004b). While this approach successfully incorporates a measure of cumulative reproductive success, it still relies on arbitrary age thresholds and fails to fully capture functional longevity (FL).

Several studies have incorporated the longitudinal measures fitted by random regression models into the genetic evaluation of longevity in beef cattle. Jamrozik et al. (2013) proposed evaluating repeated measures of stayability up to the second, third, and eighth calving, provided that the cow had her first calf at 2 years of age. Silva et al. (2018) and Silva et al. (2021) applied stayability to consecutive calvings in Zebu and Taurine/crossbred populations, respectively, and concluded that it could replace traditional single‐measure stayability in genetic evaluations. Although this methodology effectively resolves the issue of delayed phenotypes by assessing performance at earlier consecutive stages, it still does not fully differentiate cows for sexual precocity and cumulative productivity.

More recently, Oliveira et al. (2020) proposed an improved method to evaluate FL. The trait was defined as the period during which a cow remained alive and continued to calve after her first calving. In that framework, each cow was assigned a binary indicator for each year between ages 2 and 15, reflecting whether she produced a calf in that year. These longitudinal binary records were subsequently modelled through a random regression approach. By shifting the independent variable of the regression from calving order (Jamrozik et al. 2013) to age, the FL trait, as defined by Oliveira et al. (2020), effectively differentiates cows based on their long‐term productivity.

FL is a composite trait that integrates attributes directly contributing to the successful retention and productivity of a cow within the herd (Tsuruta et al. 2005). Consequently, it intrinsically incorporates multiple economically relevant traits, such as fertility, maternal ability, and health (Martinez et al. 2004a). The potential inclusion of FL in selection indices to replace traditional single‐measure stayability definitions requires reliable estimates of its genetic associations with these primary traits. Therefore, the objectives of this study were to estimate the variance components for two definitions of FL and to evaluate their genetic associations with sexual precocity and growth traits in the Nellore breed.

## 2 Material and Methods

### 2.1 Data

The dataset used in this study comprised information managed and provided by the Geneplus/Embrapa Breeding Program. Pedigree and phenotypic records of Nellore beef cattle born between 1950 and 2023, distributed across various regions of Brazil, were available. The original dataset contained 2,607,400 calving records from 814,630 cows, sourced from 787 farms distributed across the five geographical macro‐regions of Brazil. This dataset predominantly represents the primary beef‐producing areas in the country, with the majority of records originating from the Midwest (453 farms and 507,673 cows; approximately 62.3%) and Southeast (170 farms and 179,258 cows; approximately 22.0%) regions. The remaining data were sourced from the North (60 farms and 61,727 cows; approximately 7.6%), South (31 farms and 35,180 cows; approximately 4.3%), and Northeast (73 farms and 30,792 cows; approximately 3.8%) regions.

The traits analysed were age at first calving (AFC), weaning weight (WW), yearling weight (YW), and FL. FL was evaluated from 27 to 135 months of age, with phenotypes generated every 12 months, using two alternative approaches: (1) the ability to remain in the herd, evaluated as a binary trait, assigning 1 for the presence of a calving record and 0 for its absence within each interval between predefined standard ages (FLSTAY), similar to the method used by Oliveira et al. (2020); and (2) the cumulative number of calvings up to the predefined standard ages (FLNC). The age scale for the FL traits was expressed in months primarily to facilitate the biological and practical interpretation of reproductive benchmarks for both researchers and livestock producers. As orthogonal Legendre polynomials involve a standard algebraic normalization of the independent variable (rescaling ages from −1 to +1), the choice of the time unit (whether days, months, or years) is mathematically invariant and does not alter the estimated covariance functions or genetic parameters.

Preliminary analyses were performed for all traits for quality control. Only cows that calved for the first time between 20 and 51 months of age, with a calving age between 20 and 240 months, and a calving interval ranging from 290 to 820 days were retained. Cows subjected to embryo transfer and/or in vitro fertilization at any point in their lives were excluded from the study.

For WW and YW, records within the age intervals of 165 to 255 days and 505 to 595 days, respectively, were utilized. A single weight measurement per animal was considered for both traits, selecting the record closest to 210 days for WW and 550 days for YW. Furthermore, phenotypes outside the range determined by the mean plus or minus 3.5 standard deviations were excluded, as were records from contemporary groups (CGs) with fewer than three animals.

For FLSTAY and FLNC, young cows that had not yet had the opportunity to express the complete phenotype had their phenotypic vectors adjusted according to the maximum possible age at the time of data extraction. Cows that failed to calve for the first time by 51 months of age were excluded. Cows that failed to calve for two or more consecutive years before calving again had their subsequent calving records disregarded after these initial failures. Possible phenotypic vectors for hypothetical cows according to each definition are presented in Table 1. CGs were formed considering farm, year, and season of birth, along with the farm of first calving. CGs with fewer than three animals or without phenotypic variability were excluded.

**TABLE 1:** Phenotypic vectors for some hypothetical cows according to the trait definition: Functional longevity as stayability (FLSTAY) and functional longevity associated with the number of calvings (FLNC).

Cow	FLSTAY	FLNC	Profile description
A	[1,1,1,1,1,1,1,1,1,1]	[1,2,3,4,5,6,7,8,9,10]	Cow with calving records at all ages.
B	[1,1,1,1,1,0,1,1,1,0]	[1,2,3,4,5,5,6,7,8,8]	Cow with missed calvings at the 6th and 10th ages.
C	[1,1,0,0,0,0,0,0,0,0]	[1,2,2,2,2,2,2,2,2,2]	Cow with calvings only up to the 2nd age and more than 2 consecutive failures, failing at the 3rd and 4th ages.
D	[1,1,1,1,1]	[1,2,3,4,5]	Young cow whose maximum age corresponds to the 5th age, possessing all calving records up to that point.

Tables 2 and 3 present a descriptive summary of the datasets after quality control.

**TABLE 2:** Number of animals with phenotype (*n*), mean, standard deviation (SD), and minimum (Min) and maximum (Max) values for the traits age at first calving (AFC), weaning weight (WW), and yearling weight (YW).

Trait	*n*	Mean	SD	Min	Max
AFC (days)	345,062	1123	196.07	600	1552
WW (kg)	718,921	201.35	38.56	64	339
YW (kg)	343,725	356.45	80.94	98	645

**TABLE 3:** Number of animals with phenotype (*n*), mean, standard deviation (SD), minimum (Min), and maximum (Max) values for Functional Longevity as Stayability (FLSTAY) and Functional Longevity associated with the Number of Calvings (FLNC) according to predetermined ages.

Age (months)	FLSTAY					FLNC				
	*n*	Mean	SD	Min	Max	*n*	Mean	SD	Min	Max
27	130,086	0.11	0.32	0	1	144,191	0.10	0.30	0	1
39	129,041	0.72	0.45	0	1	143,120	0.80	0.49	0	2
51	124,342	0.57	0.50	0	1	138,340	1.36	0.52	1	3
63	117,586	0.43	0.49	0	1	132,093	1.78	0.76	1	4
75	111,507	0.34	0.47	0	1	126,033	2.12	1.05	1	5
87	105,171	0.26	0.44	0	1	119,106	2.38	1.32	1	6
99	98,019	0.20	0.40	0	1	112,119	2.59	1.58	1	7
111	91,094	0.15	0.35	0	1	104,852	2.76	1.81	1	8
123	84,072	0.11	0.31	0	1	97,472	2.88	2.01	1	9
135	76,629	0.08	0.26	0	1	89,744	2.96	2.17	1	10

### 2.2 Models

Four classes were defined for the season of birth according to the month of the year: March to May; June to August; September to November; and December to February.

The traits AFC, WW, and YW were analysed using an animal model. The systematic (fixed) effects included in the models were specific to each trait. For WW and YW: contemporary group (herd‐year‐season of birth and sex), age at recording (linear effect), and age of dam at calving (linear and quadratic effects); for AFC: contemporary group (herd‐year‐season of birth and herd at calving). As random effects, in addition to additive genetic and residual effects, maternal additive genetic and permanent environmental effects were included for WW.

For the traits FLSTAY and FLNC, the following random regression animal model was applied:

$$y_{\text{ijklt}}={\mathrm{YS}}_{\mathrm{it}}+\sum_{m=0}^{o-1}\beta_{\mathrm{km}}\varphi_{\mathrm{mt}}+\sum_{m=0}^{o-1}\alpha_{\mathrm{lm}}\varphi_{\mathrm{mt}}+\sum_{m=0}^{o-1}\delta_{\mathrm{lm}}\varphi_{\mathrm{mt}}+\varepsilon_{\text{ijklt}}$$

where $y_{\text{ijklt}}$ represents the FLSTAY or FLNC phenotype of cow *l* at age *t* (*t* = 27, …, 135 months); ${\mathrm{YS}}_{\mathrm{it}}$ is the systematic effect of the *i*‐th year‐season of birth subclass at age *t*; $\beta_{\mathrm{km}}$ is the *m*‐th regression coefficient for the random effect of contemporary group *k* (herd‐year‐season of birth and herd at first calving); $\alpha_{\mathrm{lm}}$ and $\delta_{\mathrm{lm}}$ are the *m*‐th regression coefficients for the random additive genetic and permanent environmental effects of cow *l*, respectively; $\varphi_{\mathrm{mt}}$ is the *m*‐th Legendre polynomial evaluated at age *t*; and $\varepsilon_{\text{ijklt}}$ is the random residual effect associated with each phenotypic observation. The random regression models were fitted using orthogonal Legendre polynomials of degree 2 (*o* = 3 coefficients) for FLNC and degree 3 (*o* = 4 coefficients) for FLSTAY. These polynomial degrees were determined in preliminary analyses based on goodness of fit and model parsimony using the Deviance Information Criterion (DIC). Additionally, cross‐validation evaluated the stability and predictive ability of the estimated breeding values (EBVs) for sires and cows across different polynomial orders. For each trait, the same degree was used for contemporary group, additive genetic, and permanent environmental effects, so that each effect had the same opportunity to explore the spaces of the curves (Jamrozik et al. 2013). A heterogeneous residual variance structure was adopted, assigning a specific residual variance to each age.

The general model used for analysis of the traits AFC and YW can be described in matrix form as follows: y=Xb+Za+e, where y is the vector of observations; **b** is the vector of systematic effects; **a** is the vector of random additive genetic effects; **e** is the vector of the random residual effect, and **X** and **Z** are incidence matrices corresponding to the observations for these effects, respectively. For WW, the basic model was modified to include the **Z**_**m**_ **a**_**m**_ and **W**_**m**_ **p**_**m**_ terms, where **a**_**m**_ and **p**_**m**_ are the vectors of random maternal additive genetic and maternal permanent environment effects, and **Z**_**m**_ and **W**_**m**_ are incidence matrices corresponding to the observations for these effects, respectively. For FLSTAY and FLNC, the model can be written as: $y=\mathrm{Xb}+\mathrm{Cg}+Za_{\mathrm{rr}}+\mathrm{Wp}+e,$ where **y** is the vector of observations; **b** is the vector of systematic effect *YS*; **g**, **a**_**rr**_, **p** and **e** are the vectors of random contemporary group, additive genetic, permanent environmental, and residual effects, respectively; **X**, **C**, **Z**, and **W** are incidence matrices corresponding to the observations for systematic, contemporary group, additive genetic, and permanent environmental effects, respectively.

The following assumptions were made for these models:

$$y|b,a,\sigma_{e}^{2}\sim \mathrm{NMV}\left(\mathrm{Xb}+\mathrm{Za},{I\sigma }_{e}^{2}\right),\mathrm{Var}\left[\begin{array}{c} a\\ e \end{array}\right]=\left[\begin{array}{cc} {A\sigma }_{a}^{2} & 0\\ 0 & {I\sigma }_{e}^{2} \end{array}\right]$$

$$y|b,a,a_{m},p_{m},\sigma_{e}^{2}\sim \mathrm{NMV}\left(\mathrm{Xb}+\mathrm{Za}+Z_{m}a_{m}+W_{m}p_{m},{I\sigma }_{e}^{2}\right),\mathrm{Var}\left[\begin{array}{c} a\\ a_{m}\\ p_{m}\\ e \end{array}\right]=\left[\begin{array}{cccc} {A\sigma }_{a}^{2} & 0 & 0 & 0\\ 0 & {A\sigma }_{\mathrm{am}}^{2} & 0 & 0\\ 0 & 0 & {I\sigma }_{\mathrm{pm}}^{2} & 0\\ 0 & 0 & 0 & {I\sigma }_{e}^{2} \end{array}\right]$$

$$y|b,g,a_{\mathrm{rr}},p,R\sim \mathrm{NMV}\left(\mathrm{Xb}+\mathrm{Cg}+Za_{\mathrm{rr}}+\mathrm{Wp},R\right),\mathrm{Var}\left[\begin{array}{c} g\\ a_{\mathrm{rr}}\\ p\\ e \end{array}\right]=\left[\begin{array}{cccc} \Lambda_{G}⨂I & 0 & 0 & 0\\ 0 & \Lambda_{A}⨂A & 0 & 0\\ 0 & 0 & \Lambda_{P}⨂I & 0\\ 0 & 0 & 0 & R \end{array}\right]$$

where $\sigma_{a}^{2}$, $\sigma_{\mathrm{am}}^{2}$, $\sigma_{\mathrm{pm}}^{2}$ and $\sigma_{e}^{2}$ are, respectively, the direct additive genetic, maternal additive genetic, maternal permanent environmental and residual variances; $\Lambda_{G}$, $\Lambda_{A}$ and $\Lambda_{P}$ are contemporary group, additive genetic, and permanent environmental covariance matrices between the regression coefficients, respectively; **R** is the 10 × 10 diagonal residual variance matrix, with one specific variance for each age; **A** is the numerator relationship matrix between animals; **I** is an identity matrix, and ⊗ is the direct product operator between matrices.

### 2.3 Analyses

The analyses were performed using a Bayesian method with the GIBBSF90+ software (Lourenco et al. 2022). The following prior distributions were assumed for the model:

b∝constant,

$$a\;|\;A,\sigma_{a}^{2}\sim N\left(0,{A\sigma }_{a}^{2}\right),$$

$$a_{m}\;|\;A,\sigma_{\mathrm{am}}^{2}\sim N\left(0,{A\sigma }_{\mathrm{am}}^{2}\right),$$

$$p_{m}\;|\;\sigma_{\mathrm{pm}}^{2}\sim N\left(0,{I\sigma }_{\mathrm{pm}}^{2}\right),$$

$$g\;|\;\Lambda_{G}\sim \mathrm{MVN}\left(0,\Lambda_{G}⊗I\right),$$

$$a_{\mathrm{rr}}\;|\;\Lambda_{A}\sim \mathrm{MVN}\left(0,\Lambda_{A}⊗A\right),$$

$$p\;|\;\Lambda_{P}\sim \mathrm{MVN}\left(0,\Lambda_{P}⊗I\right),$$

$$\sigma_{a}^{2}\;|\;v_{a},s_{a}^{2}\sim \mathrm{SIC}\left(v_{a},v_{a}s_{a}^{2}\right),$$

$$\sigma_{\mathrm{am}}^{2}\;|\;v_{\mathrm{am}},s_{\mathrm{am}}^{2}\sim \mathrm{SIC}\left(v_{\mathrm{am}},v_{\mathrm{am}}s_{\mathrm{am}}^{2}\right),$$

$$\sigma_{\mathrm{pm}}^{2}\;|\;v_{\mathrm{pm}},s_{\mathrm{pm}}^{2}\sim \mathrm{SIC}\left(v_{\mathrm{pm}},v_{\mathrm{pm}}s_{\mathrm{pm}}^{2}\right),$$

$$\sigma_{e}^{2}\;|\;v_{e},s_{e}^{2}\sim \mathrm{SIC}\left(v_{e},v_{e}s_{e}^{2}\right),$$

$$\Lambda_{G}\;|\;v_{g},S_{g}\sim \mathrm{IW}\left(v_{g},v_{g}S_{g}\right),$$

$$\Lambda_{A}\;|\;v_{\mathrm{arr}},S_{\mathrm{arr}}\sim \mathrm{IW}\left(v_{\mathrm{arr}},v_{\mathrm{arr}}S_{\mathrm{arr}}\right),$$

$$\Lambda_{P}\;|\;v_{p},S_{p}\sim \mathrm{IW}\left(v_{p},v_{p}S_{p}\right),$$

$$R\;|\;v_{e},S_{e}\sim \mathrm{IW}\left(v_{e},v_{e}S_{e}\right),$$

where *N*, *MVN*, *SIC* and *IW* indicate normal, multivariate normal, scaled inverted chi‐square and inverse Wishart distributions, respectively, and $v_{a},s_{a}^{2}$, $v_{\mathrm{am}},s_{\mathrm{am}}^{2}$, $v_{\mathrm{pm}},s_{\mathrm{pm}}^{2}$, $v_{e},s_{e}^{2}$, $v_{g},S_{g}$, $v_{\mathrm{arr}},S_{\mathrm{arr}}$, $v_{p},S_{p}$, and $v_{e},S_{e}$ correspond to the degree of confidence and prior values for the variance or covariance matrix according to the effect.

Covariance components were estimated via single‐trait analyses. A single chain of 300,000 iterations was generated for each trait, with an initial burn‐in period of 100,000 iterations and a thinning interval of 10. Chain convergence was assessed through visual inspection of trace plots across iterations, as well as by the criteria of Geweke (1991) and Raftery and Lewis (1992). Thus, the variance components and genetic parameters (heritability and genetic correlation) were estimated from the remaining samples.

Based on the posterior mean estimates for the (co)variance components, the EBVs of the animals were predicted for all studied traits using the BLUPF90+ software (Lourenco et al. 2022).

For FLSTAY and FLNC, the estimates for (co)variance components, heritability, repeatability and breeding values (EBVs) on the longitudinal scale were calculated using the standard formulae of linear functions of regression coefficients as proposed by (Jamrozik and Schaeffer 1997). For both FLSTAY and FLNC, longitudinal heritabilities at each specific age were calculated as the ratio of additive genetic variance to the phenotypic variance (i.e., the sum of contemporary group, additive genetic, permanent environmental, and residual variances). Similarly, longitudinal repeatability estimates were calculated as the ratio of the sum of additive genetic and permanent environmental variances to the phenotypic variance. The accuracies of the EBVs were obtained using the formula $\sqrt{\frac{1-\mathrm{PEV}}{\sigma_{\alpha }^{2}}}$, where PEV is the prediction error variance and $\sigma_{\alpha }^{2}$ is the additive genetic variance for the trait.

The method proposed by Calo et al. (1973) was utilized to estimate the genetic correlations between FLSTAY and FLNC with other traits, as employed in several other studies (e.g., Dikmen et al. 2012; Luo et al. 2021). Approximate genetic correlations were calculated from the EBVs of individuals with an accuracy of at least 0.60 for both traits. The following formulas were used:

$${\hat{r}}_{g1,2}=\frac{\sqrt{\sum {\mathrm{AC}}_{1}^{2}\times \sum {\mathrm{AC}}_{2}^{2}}}{\sum \left({\mathrm{AC}}_{1}^{2}\times {\mathrm{AC}}_{2}^{2}\right)}\times r_{1,2},\mathrm{SE}=\sqrt{\frac{1-{\hat{r}}_{g1,2}^{2}}{n-2}},$$

where ${\hat{r}}_{g1,2}$ is the approximate genetic correlation between traits 1 and 2; AC_1_ and AC_2_ are accuracies of traits 1 and 2; $r_{1,2}$ is the Pearson correlation between EBVs for traits 1 and 2; *n* is the number of animals (Sokal and Rohlf 1995); and SE is the standard error of ${\hat{r}}_{g1,2}$, used to calculate the 95% confidence interval.

The choice of Calo's method to estimate approximate genetic correlations, rather than multiple‐trait analyses, was driven by the structural complexity and parameterization required to combine these traits. Functional longevity was modelled as a longitudinal trait via random regression, which partitions the non‐genetic variance into contemporary group trajectories, permanent environmental trajectories, and heterogeneous residual variances across 10 age classes. In contrast, the evaluated reproductive (AFC) and growth (WW and YW) traits are single‐measure phenotypes, with WW additionally encompassing maternal effects. Accurately modelling the environmental covariance between a longitudinal trajectory and a single time‐point record presents a structural limitation, often leading to non‐convergence or biased parameter estimates. Consequently, Calo's method was adopted as a computationally feasible alternative. In this case, this approach enables a more accurate assessment of the underlying genetic associations based on highly reliable EBVs, avoiding the confounding artefacts of misspecified environmental covariance structures.

## 3 Results and Discussion

The mean and variability for AFC, WW, and YW (Table 2) were similar to those reported in previous studies using data subsets from Nellore cattle in Brazil (Mota et al. 2024; Pereira et al. 2016; Ramos et al. 2021). To better characterize the phenotypic variability and the timing of first reproductive success within the evaluated population, the cumulative distribution of AFC was summarized. Within the analysed dataset, 10.37% (35,787 cows) calved for the first time before 27 months of age. This proportion establishes the phenotypic differentiation (score 1 versus 0) for the FL traits at this initial threshold. By 39 months of age, this proportion increased substantially to 74.70% (257,763 cows), encompassing the majority of the herd's first reproductive cycle. By methodological definition, 100% of the retained females (345,062 cows) had calved by the maximum limit of 51 months. The restricted proportion of early‐calving females (10.37% at 27 months) reflects the biological and management realities of Zebu cattle raised under pasture conditions, suggesting that phenotypic success at this early age is a challenging benchmark characterizing sexual precocity.

For FLSTAY, the number of animals with recorded phenotypes at each age was typically lower than that for FLNC (Table 3). This discrepancy arises from the CGs filtering process. Due to the nature of phenotypic data recording, CGs in which all cows had a phenotype of 0 (or all equaled 1) for FLSTAY exhibited no phenotypic variability and were consequently excluded from the analysis. Conversely, for FLNC, these CGs could still display phenotypic variation and were therefore retained in the analysis.

When examining the phenotypic means for FLSTAY and FLNC across different ages, it is crucial to account for the methodological penalty applied to both traits: for cows that did not calve for two consecutive years, any subsequent calving records were disregarded. While this penalization mimics practical management strategies where unproductive cows are discarded, it introduces a limitation inherent to large‐scale commercial datasets: the potential confounding between true biological reproductive failure, voluntary culling decisions (e.g., sales, structural unsoundness, or death) and even recording omissions. To overcome this confounding effect and enhance the accuracy of future genetic evaluations for FL, breed associations must prioritize implementing comprehensive recording systems that specifically document the reasons for animal disposal. Under this constraint, FLSTAY exhibited a peak success rate at 39 months of age (0.72), indicating that the majority of cows had their first calf by this age. Following this peak, the mean gradually declined to 0.08 at 135 months. This substantial reduction likely reflects the gradual culling of animals and the permanent assignment of failure due to consecutive reproductive gaps. Conversely, the mean for FLNC increased steadily throughout the productive life of the cow, as is expected for a cumulative trait. However, by 135 months of age, the mean number of accumulated calvings was only 2.96. Considering the biological potential for up to ten calvings within this timeframe, this relatively low average highlights the impact of reproductive failures (which lead to the methodological plateau effect) and possibly a high culling rate in the evaluated Nellore population.

### 3.1 Heritabilities

Generally, weight traits exhibited low to moderate heritability estimates (Table 4), whereas AFC (Table 4), FLSTAY, and FLNC (Table 5) presented lower heritability estimates.

**TABLE 4:** Means, standard deviations (SD), and 95% highest posterior density intervals (HPD 95%) for the heritabilities of the traits age at first calving (AFC), weaning weight—direct effect (WWD), weaning weight—maternal effect (WWM), and yearling weight—550 days (YW) in Nellore cattle.

Trait	Mean	SD	HPD 95%
AFC	0.07	0.003	0.07–0.08
WW_D_	0.16	0.003	0.15–0.17
WW_M_	0.06	0.001	0.05–0.07
YW	0.30	0.005	0.29–0.31

**TABLE 5:** Heritability estimates (95% highest posterior density intervals – HPD95) for functional longevity as stayability (FLSTAY) and functional longevity associated with the number of calvings (FLNC) according to the predetermined age in Nellore cattle.

Age (months)	FLSTAY			FLNC		
	Mean	SD	HPD 95%	Mean	SD	HPD 95%
27	0.020	0.002	0.017–0.023	0.022	0.002	0.018–0.026
39	0.004	0.000	0.004–0.005	0.020	0.001	0.018–0.022
51	0.012	0.001	0.010–0.014	0.044	0.003	0.039–0.049
63	0.022	0.002	0.019–0.025	0.060	0.004	0.054–0.067
75	0.031	0.002	0.027–0.035	0.070	0.004	0.063–0.078
87	0.038	0.002	0.033–0.043	0.074	0.004	0.067–0.083
99	0.042	0.003	0.037–0.048	0.075	0.004	0.067–0.084
111	0.045	0.003	0.039–0.051	0.075	0.004	0.067–0.083
123	0.042	0.003	0.035–0.048	0.074	0.004	0.065–0.081
135	0.033	0.003	0.027–0.039	0.070	0.004	0.062–0.077

The low heritability estimate for AFC indicates the predominance of environmental effects on this trait, which is consistent with previous studies conducted on Nellore cattle (Kluska et al. 2018; Morales et al. 2024; Pereira et al. 2016). Conversely, some studies have reported higher heritability estimates (Eler et al. 2014; Ramos et al. 2021). Heritability estimates for AFC can be influenced by the implementation or absence of breeding seasons on farms (Ramos et al. 2021). The proportion of farms utilizing a breeding season can vary substantially among different data subsets within the same breed. Furthermore, the dataset used in the present study is extensive and contains farms located in diverse regions of Brazil, which employ a variety of management practices. This variation could inflate the residual variance for AFC, consequently reducing the heritability estimate for the trait.

For WW, the heritability estimates for direct and maternal effects fell within the ranges reported in the literature for the Nellore breed, although they were lower than those reported in some studies (Carvalho et al. 2025; Kluska et al. 2018; Morales et al. 2024; Pereira et al. 2016). The moderate heritability estimate observed for YW confirms its greater potential for response to selection and aligns with values reported in other studies (Morales et al. 2024; Pereira et al. 2016; Ramos et al. 2021). However, some authors have reported higher estimates (Carvalho et al. 2025; Schmidt et al. 2019), particularly when incorporating genomic information into the analyses (Kluska et al. 2018). Overall, these results support the consensus that growth traits can yield significant genetic progress when subjected to selection.

Heritability estimates for FLNC were consistently higher than those for FLSTAY (Table 5). These findings suggest that selecting for FL could be effective due to the genetic variation present among animals; however, genetic improvement would be achieved over the long term. The heritability estimates for FLSTAY are lower than those documented for stayability to consecutive calvings in Zebu (Silva et al. 2018) and Taurine (Jamrozik et al. 2013; Silva et al. 2021) populations. Passafaro et al. (2024), utilizing an FL definition similar to stayability to consecutive calvings (Jamrozik et al. 2013), observed heritability estimates declining from 0.16 to 0.04 in a multibreed cattle population. For an FL definition comparable to FLSTAY, Oliveira et al. (2020) reported heritability estimates ranging from 0.07 to 0.25, whereas Aliloo et al. (2025) found estimates generally below 0.10 in North American and Australian Angus cattle, respectively. Investigating a multibreed population, Brzáková et al. (2019) reported heritability estimates below 0.14 for FL, defined as the number of calves produced by four specific ages (78, 90, 150, and 160 months) and analysed using single‐trait or multiple‐trait models.

Traits related to survival, longevity, and herd stayability tend to exhibit lower heritabilities due to their strong dependence on multiple environmental factors and management decisions. Nevertheless, even low heritabilities are sufficient to enable long‐term genetic progress, particularly when integrating phenotypes, genomic information, and data from traits correlated with longevity.

### 3.2 Repeatabilities

Repeatability estimates for FLNC were consistently higher than those for FLSTAY (Table 6), starting at 0.13 at 27 months and rapidly increasing to a plateau around 0.80 at older ages. In contrast, FLSTAY repeatability estimates ranged from 0.03 to 0.57. The moderate to high repeatability estimates observed for FLNC, particularly from 51 months onwards, highlight the substantial impact of permanent animal effects (both genetic and non‐genetic) on a cow's cumulative productivity. The repeatability estimates and their trend for FLSTAY are similar to those reported for stayability to consecutive calvings in Zebu (Silva et al. 2018) and Taurine (Jamrozik et al. 2013; Silva et al. 2021) populations.

**TABLE 6:** Repeatability estimates (95% highest posterior density intervals – HPD95) for functional longevity as stayability (FLSTAY) and functional longevity associated with the number of calvings (FLNC) according to the predetermined age in Nellore cattle.

Age (months)	FLSTAY			FLNC		
	Mean	SD	HPD 95%	Mean	SD	HPD 95%
27	0.066	0.003	0.061–0.070	0.128	0.007	0.114–0.142
39	0.031	0.001	0.030–0.032	0.226	0.002	0.223–0.230
51	0.106	0.001	0.104–0.109	0.527	0.002	0.522–0.531
63	0.205	0.002	0.202–0.209	0.704	0.003	0.698–0.710
75	0.307	0.002	0.302–0.311	0.787	0.004	0.779–0.794
87	0.402	0.002	0.397–0.407	0.802	0.004	0.794–0.810
99	0.469	0.003	0.463–0.474	0.795	0.004	0.787–0.804
111	0.507	0.004	0.500–0.514	0.793	0.005	0.784–0.802
123	0.509	0.003	0.502–0.516	0.793	0.005	0.783–0.802
135	0.572	0.006	0.560–0.584	0.774	0.005	0.764–0.784

The repeatability estimates for FLSTAY and FLNC should be interpreted with caution. Understanding the longitudinal dynamics of these parameters (variances and variance ratios) requires combining statistical behaviour with biological and management aspects. In commercial beef operations, remaining in the herd is not merely a random survival event. Instead, it reflects a continuous, latent selection index imposed by the breeder, where cows must repeatedly overcome successive biological and economic obstacles, such as regular fertility, adequate maternal ability, and physical soundness (Brzáková et al. 2019; Catrett et al. 2024). Consequently, phenotypes at advanced ages capture the entire history of a cow's performance. This cumulative biological filtering could explain the pronounced expansion of the permanent environmental variance, and thus the repeatability, at later ages. From a mathematical standpoint, once a female is culled or penalized for consecutive reproductive failures, no novel phenotypic information is introduced to alter her phenotypic trajectory. The random regression framework manages this longitudinal redundancy by absorbing the locked, parallel trajectories of late‐stage records into the permanent environmental component, thereby isolating this scale and preventing the artificial inflation of the heritabilities.

### 3.3 Genetic Correlations

The estimates for the approximate genetic correlations between FLSTAY and FLNC were consistently high (≈0.90) across all evaluated ages (Figure 1). This result was expected, considering that, in essence, the two traits represent alternative and similar approaches to the same selection objective. A cow that calves regularly (which determines success in FLSTAY) will naturally accumulate more calvings up to a specific age in FLNC.

**FIGURE 1:**
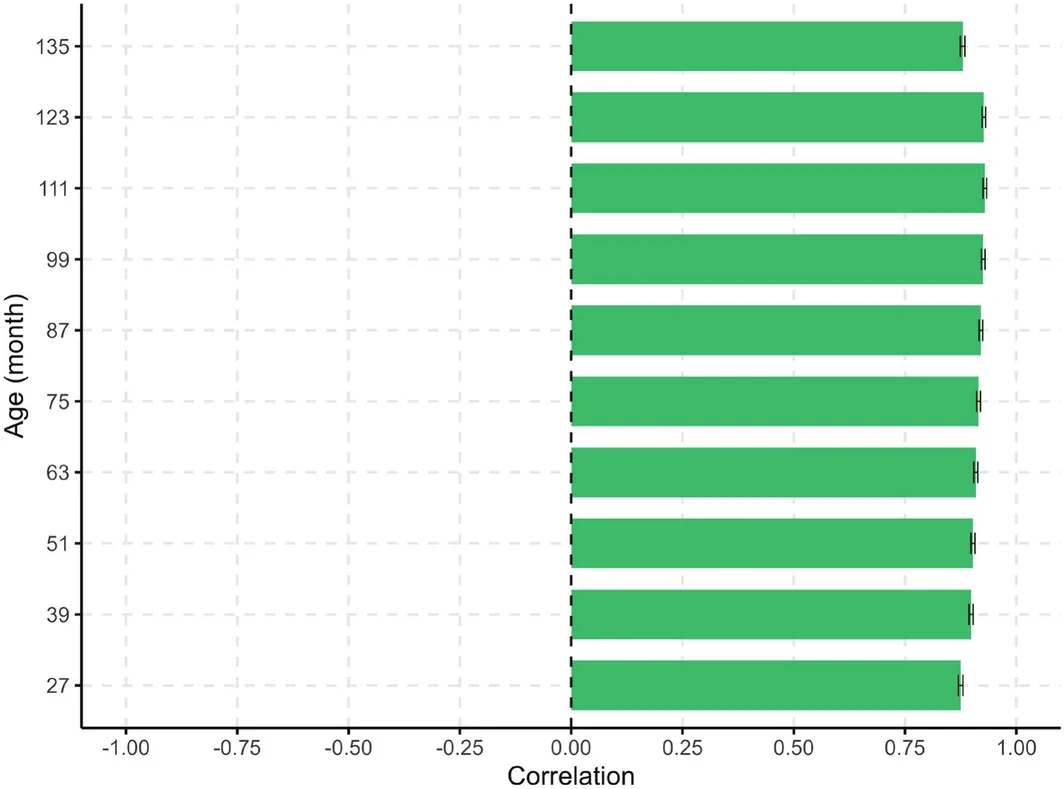
Approximate genetic correlations and confidence intervals (CI 95%) between functional longevity as stayability (FLSTAY) and functional longevity associated with the number of calvings (FLNC) according to the predetermined age in Nellore cattle. [Colour figure can be viewed at wileyonlinelibrary.com]

The approximate genetic correlation estimates between AFC and both FLSTAY and FLNC were negative across all ages (Figure 2). The correlations were high at 27 months of age (≈−0.75) and gradually decreased to values around −0.25 from 63 months onwards. It is important to note that remaining in the herd (which, in our study, determines calving success and affects both FLSTAY and FLNC) is an outcome that depends, at least partially, on voluntary culling decisions made by the breeder and is subject to the specific selection criteria of each farm (Morales et al. 2024; Silva et al. 2021). Regarding AFC, it is reasonable to expect that the genetic potential for this trait would favourably impact FLSTAY and FLNC, as sexual precocity is likely a relevant selection criterion for the majority of breeders. Additionally, an earlier first calving can positively impact FLNC phenotypes, particularly at the earlier evaluated ages. The gradual reduction in correlation estimates with advancing age could be justified by the natural decrease in the relevance of AFC to FLSTAY and FLNC at older ages, when regular calving frequency becomes more important. Morales et al. (2024), studying stayability to consecutive calvings in Taurine and Zebu cattle, observed favourable but lower magnitude correlations between AFC and stayability, which however exhibited the same decreasing trend with increasing parity order. In a study involving Limousin cattle in France, Callegaro et al. (2024) reported negative genetic correlation estimates (−0.07 to −0.26) between AFC and stayability to consecutive calvings, showing an increasing trend across parities. Additionally, the high‐magnitude and favourable genetic correlation estimate at 27 months of age (−0.75) highlights a promising perspective from a practical standpoint. In Zebu cattle, first calving at 27 months is a challenging benchmark characterizing sexual precocity. Since the phenotypic expression for both FL traits at this initial age is restricted to strictly binary outcomes (0 for failure, 1 for success), the resulting EBVs at 27 months mathematically translate to the animal's genetic merit for the probability of precocious calving. Consequently, FL evaluated at early ages could serve as a direct indicator or auxiliary selection criterion to improve sexual precocity. A major advantage of this random regression framework is that it empowers breeders to simultaneously select for early reproductive success (using EBVs at 27 months) and long‐term cumulative productivity (using EBVs at advanced ages chosen at the discretion of the breeding program) within a single genetic evaluation. Other studies in the literature, using single‐measure animal models fitting the traditional definition of stayability (where the target age is approximately 76 months) in the Nellore breed, observed genetic correlation estimates between AFC and stayability ranging from −0.15 to −0.64 (Costa et al. 2020; Eler et al. 2014; Kluska et al. 2018; Schmidt et al. 2019). In our study, the age closest to the traditional stayability definition was 75 months, for which the correlation between AFC and FLSTAY was −0.22, and between AFC and FLNC was −0.28. Despite the differences in trait definitions between the studies, a reasonable concordance between the estimates of genetic correlation can be observed.

**FIGURE 2:**
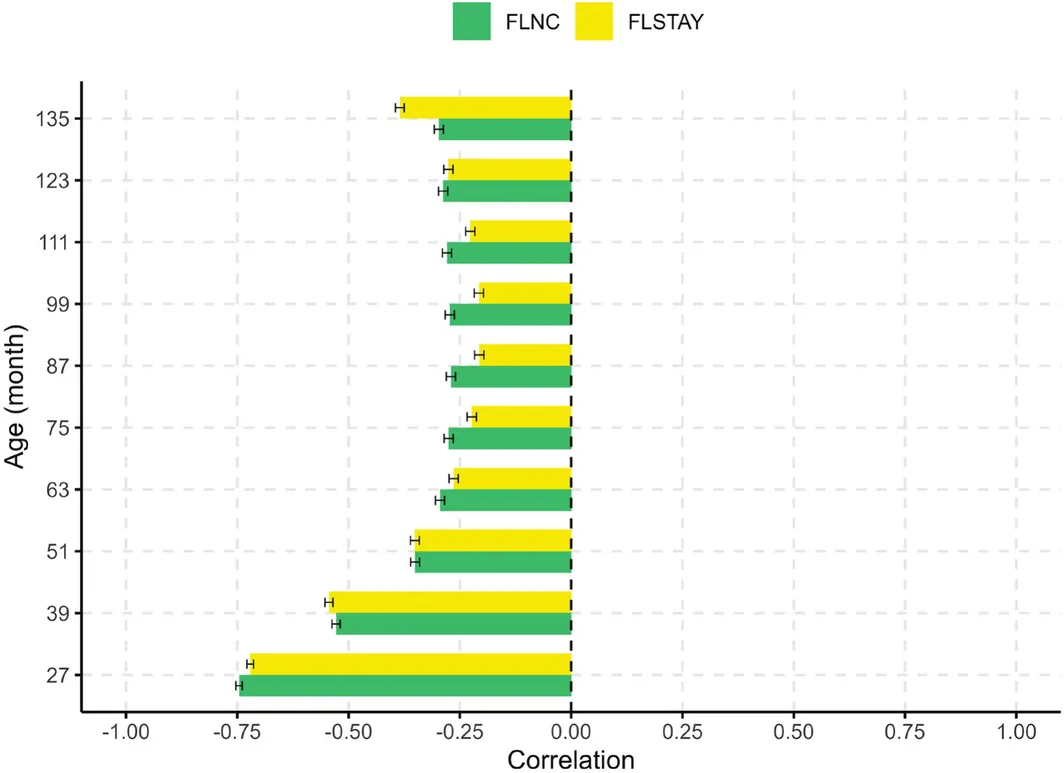
Approximate genetic correlations and confidence intervals (CI 95%) between age at first calving and functional longevity as stayability (FLSTAY) or functional longevity associated with the number of calvings (FLNC) according to the predetermined age in Nellore cattle. [Colour figure can be viewed at wileyonlinelibrary.com]

The approximate genetic correlation estimates between WW (direct effect) and both FLSTAY and FLNC were positive across all ages (Figure 3). The correlations were high at 27 months (≈0.58) and gradually decreased to values around 0.06 for FLNC (at 75 months) and 0.15 for FLSTAY (at 87 months), before increasing slightly at more advanced ages. The approximate genetic correlation estimates between WW (maternal effect) and both FLSTAY and FLNC were also positive (≈0.20) across all ages (Figure 4), although slightly lower at 27 months (≈0.10). The moderate to high correlation (WW—direct effect) observed at 27 months of age may reflect a favourable genetic association between WW and reproductive precocity and, at least partially, the preference of breeders in selecting heavier females to retain in the herd. Boligon and Albuquerque (2011), studying the Nellore breed, reported a genetic correlation estimate of 0.19 between heifer pregnancy at 16 months of age and weight gain from birth to weaning. Morales et al. (2024) reported favourable correlations for WW (both direct and maternal effects) and stayability to consecutive calvings, showing a decreasing trend until the 5th calving in Nellore cattle. However, the authors found negative correlations from the 6th calving onwards. Pereira et al. (2025) evaluated different definitions of stayability according to the minimum number of calvings at various ages in the Nellore breed and found genetic correlation values between WW (direct effect) and stayability ranging from 0.17 to 0.29. Thus, these results indicate that WW has a weak but favourable genetic relationship with functional longevity.

**FIGURE 3:**
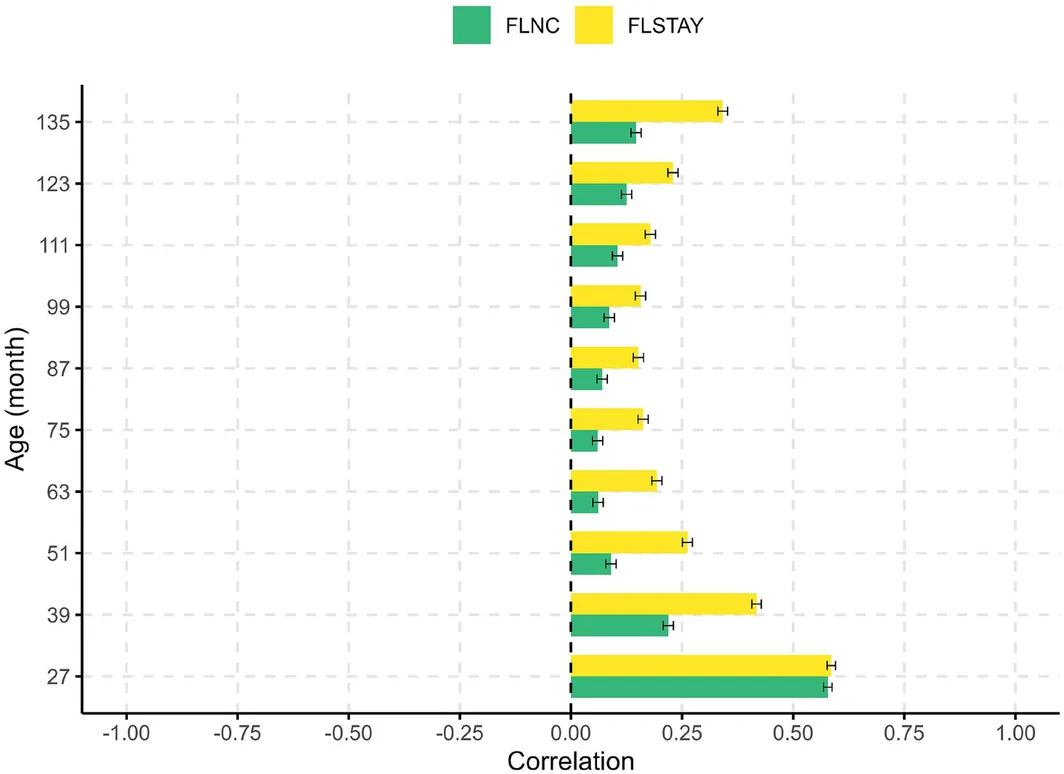
Approximate genetic correlations and confidence intervals (CI 95%) between weaning weight (direct effect) and functional longevity as stayability (FLSTAY) or functional longevity associated with the number of calvings (FLNC) according to the predetermined age in Nellore cattle. [Colour figure can be viewed at wileyonlinelibrary.com]

**FIGURE 4:**
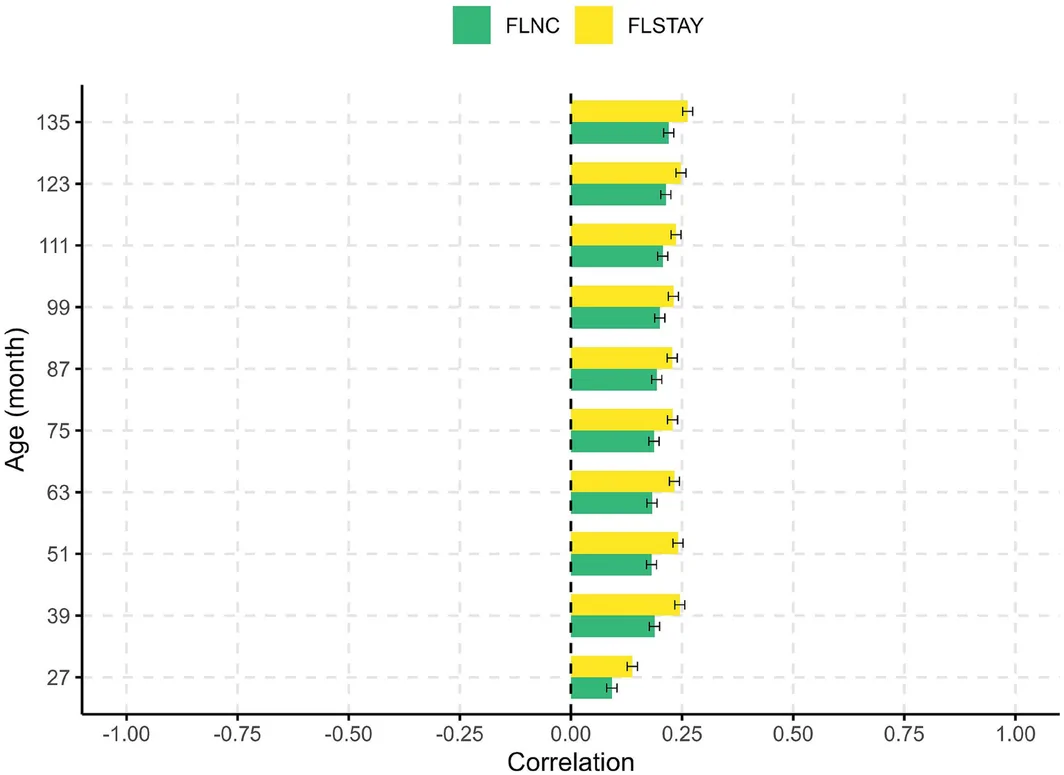
Approximate genetic correlations and confidence intervals (CI 95%) between weaning weight (maternal effect) and functional longevity as stayability (FLSTAY) or functional longevity associated with the number of calvings (FLNC) according to the predetermined age in Nellore cattle. [Colour figure can be viewed at wileyonlinelibrary.com]

The approximate genetic correlation estimates between YW and both FLSTAY and FLNC were positive across all ages (Figure 5) and exhibited the same trend as the WW (direct effect) results. The correlations were somewhat higher at 27 months (≈0.28) and gradually decreased to values around 0.10 near 75 months, returning to slightly higher values at more advanced ages. Mota et al. (2024) evaluated sexual precocity at 14 and 18 months of age in Nellore cattle and reported genetic correlation estimates with YW of 0.25 and 0.29, respectively. Similarly, Boligon and Albuquerque (2011) reported a genetic correlation estimate of 0.25 between heifer pregnancy at 16 months of age and YW. Morales et al. (2024) reported favourable correlations for YW and stayability to consecutive calvings, with a decreasing trend until the 5th calving and unfavourable correlations from the 6th calving onwards. Other studies on the Nellore breed have reported genetic correlation estimates between YW and stayability ranging from 0.18 to 0.34 (Kluska et al. 2018; Pereira et al. 2025). As with WW, these results indicate that YW has a weak but favourable genetic relationship with functional longevity.

**FIGURE 5:**
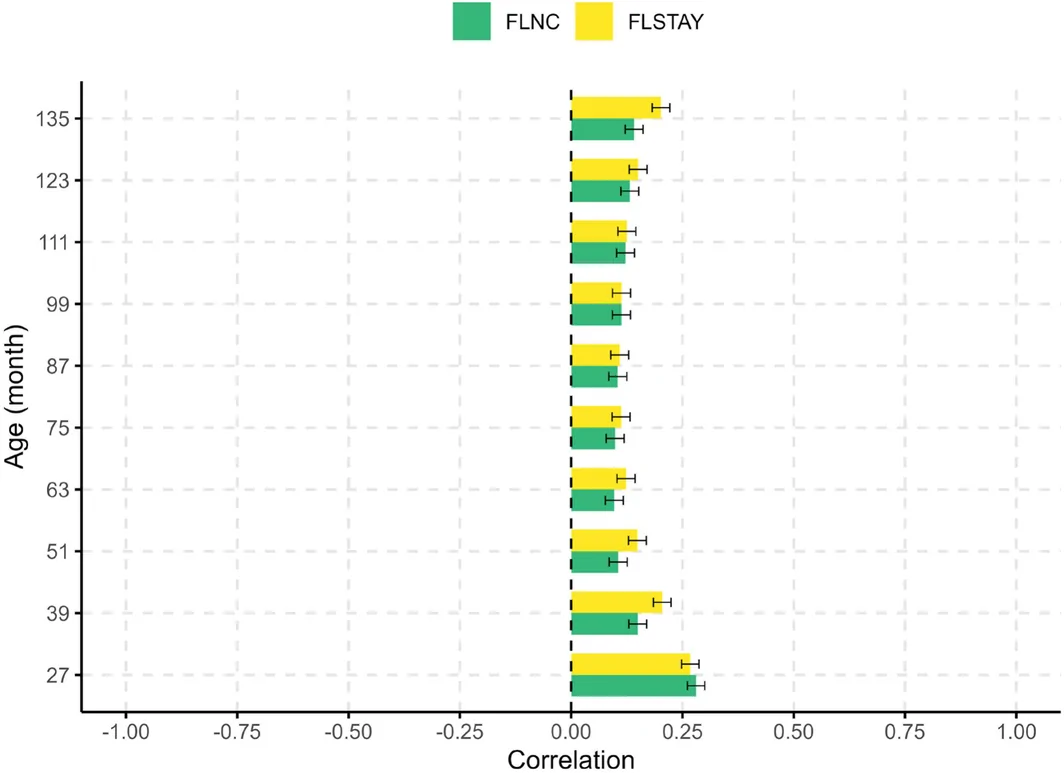
Approximate genetic correlations and confidence intervals (CI 95%) between yearling weight (at 550 days) and functional longevity as stayability (FLSTAY) or functional longevity associated with the number of calvings (FLNC) according to the predetermined age in Nellore cattle. [Colour figure can be viewed at wileyonlinelibrary.com]

Compared to traditional stayability, which is conventionally defined as a single binary observation at a fixed age (e.g., 72 or 76 months), the FLSTAY and FLNC traits proposed herein offer practical improvements for beef cattle breeding programs. Traditional stayability evaluations require waiting several years for females to reach the target age to express the phenotype, which inherently leads to low accuracy in the genetic evaluation of young sires and increases the generation interval (Jamrozik et al. 2013). In contrast, modelling FL longitudinally allows for the inclusion of partial records from younger females, maximizing data utilization and thereby potentially improving the accuracy of EBVs for young sires. Furthermore, this longitudinal approach provides the flexibility to identify animals whose genetic merit for FL aligns with the specific economic goals of different production systems, whether emphasizing early reproductive success, sustained long‐term retention, or both. Additionally, FLSTAY and FLNC can more effectively differentiate cows based on their long‐term productivity by a specified age compared to traditional stayability.

In theory, the underlying additive genetic merit for FLNC at a given age is equivalent to the sum of the genetic merits for FLSTAY up to that same age. However, despite capturing a similar genetic background, FLNC offers an advantage in the practical interpretability of its genetic predictions in sire summaries. Since FLNC is intrinsically modelled as a cumulative trait, its expected progeny difference (EPD) is directly expressed on a tangible biological scale: the expected difference in the cumulative number of calves produced by a sire's daughters up to that specific age. In contrast, conveying the equivalent long‐term merit using FLSTAY would require summing additive genetic deviations across multiple intervals, whereas evaluating FLSTAY at a single age provides only a genetic deviation on a probability scale for retention at a specific cross‐section.

## 4 Conclusions

The evaluated functional longevity measures can be readily implemented in routine genetic evaluations. However, FLNC should be preferred over FLSTAY as a selection criterion due to its transparent interpretability, less‐parameterized model, and consistently higher heritability estimates.

The estimated genetic associations were consistently favourable for both FLSTAY and FLNC. Therefore, there is no genetic antagonism between functional longevity and the growth or sexual precocity traits conventionally considered in beef cattle selection. These findings support updating current selection indices in breeding programs to include functional longevity, which offers a feasible alternative to the traditional stayability trait.

## Funding

This research was supported by Conselho Nacional de Desenvolvimento Científico e Tecnológico (CNPq: 312480/2021‐0) and Coordenação de Aperfeiçoamento de Pessoal de Nível Superior.

## Conflicts of Interest

The authors declare no conflicts of interest.

## Data Availability

The data that support the findings of this study are available from Geneplus/Embrapa Breeding Program. Restrictions apply to the availability of these data, which were used under licence for this study. Data are available from the author(s) with the permission of Geneplus/Embrapa Breeding Program.
